# Outcomes of Bariatric Surgery in People With Human Immunodeficiency Virus: A Retrospective Analysis From the ATHENA Cohort

**DOI:** 10.1093/cid/ciad404

**Published:** 2023-07-01

**Authors:** Leena Zino, Ferdinand Wit, Casper Rokx, Jan G den Hollander, Mark van der Valk, Olivier Richel, David M Burger, Angela Colbers

**Affiliations:** Department of Pharmacy and Radboudumc Research Institute for Medical Innovation, Radboud University Medical Center, Nijmegen, The Netherlands; Data Analysis, Reporting & Research Unit, Stichting HIV Monitoring, Amsterdam, The Netherlands; Department of Medical Microbiology and Infectious Diseases and Department of Internal Medicine, Section of Infectious Diseases, Erasmus University Medical Center, Rotterdam, The Netherlands; Department of Internal Medicine and Infectious Diseases, Maasstad ziekenhuis, Rotterdam, The Netherlands; Data Analysis, Reporting & Research Unit, Stichting HIV Monitoring, Amsterdam, The Netherlands; Department of Infectious Diseases, Amsterdam Institute for Infectious Diseases, Amsterdam University Medical Center, Amsterdam, The Netherlands; Department of Infectious Disease and Radboudumc Research Institute for Medical Innovation, Radboud University Medical Center, Nijmegen, The Netherlands; Department of Pharmacy and Radboudumc Research Institute for Medical Innovation, Radboud University Medical Center, Nijmegen, The Netherlands; Department of Pharmacy and Radboudumc Research Institute for Medical Innovation, Radboud University Medical Center, Nijmegen, The Netherlands

**Keywords:** bariatric surgery, antiretrovirals, weight, virologic failure

## Abstract

**Background:**

The implications of bariatric surgery (BS) on virologic and metabolic outcomes in people with human immunodeficiency virus (HIV; PWH) on antiretroviral therapy (ART) are unknown.

**Methods:**

Here, we report a retrospective analysis up to 18 months post-BS in PWH from the AIDS Therapy evaluation in The Netherlands (ATHENA) cohort with data from all dutch HIV treating Centers. Primary end points were a confirmed virologic failure (2 consecutive HIV-RNA measurements >200 copies/mL) and the percentage of patients who achieved >20% total body weight loss up to 18 months post-BS. Switches from baseline ART and trough plasma concentrations of antiretrovirals were also reported post-BS. Metabolic parameters and medication usage were compared pre- and post-BS.

**Results:**

Fifty-one patients were included. One case of confirmed virologic failure and 3 cases with viral blips were detected in this cohort up to 18 months post-BS. Eighty-five percent of patients achieved >20% total body weight loss at 18 months post-BS, with a mean difference from baseline (95% confidence interval) of −33.5% (−37.7% to −29.3%). Trough plasma concentrations of measured antiretroviral agents were all above minimum effective concentrations, except for 1 sample of darunavir. Lipid profiles, but not serum creatinine and blood pressure, improved significantly (*P* < .01) post-BS. Total medications and obesity-related comedications declined from 203 to 103 and from 62 to 25, respectively, at 18 months post-BS.

**Conclusions:**

BS was an effective intervention for weight loss and lipid control in PWH using ART in this cohort with no clear link to poor virologic outcomes.

## Teaser

Data from a Dutch cohort of people with HIV on antiretrovirals demonstrate that bariatric surgery is a beneficial intervention for weight loss and lipid improvement with no clear worsening of virologic control up to 18 months post-surgery.

People with human immunodeficiency virus (HIV; PWH) are showing increased obesity rates that are similar to, or higher than those of the general population [1]. In the Netherlands, the prevalence of obesity, defined as a body mass index (BMI) >30 kg/m^2^, among male PWH is close to that of the general population (11.6% vs 12.3% in the general male Dutch population), while it is doubled in female PWH (31.7%) compared with the general female Dutch population (15.4%) [2]. Particularly in PWH, obesity is associated with a greater risk of morbidity and mortality [3, 4], including a higher incidence of cardiovascular diseases, diabetes, and hyperglycemia compared with the general population [5, 6].

In addition to a sedentary lifestyle and increased dietary intake, reports suggest an additive role for some antiretrovirals, that is, integrase inhibitors (INSTIs) and tenofovir alafenamide fumarate (TAF), in the escalation of the incidence of obesity or metabolic diseases in PWH [7, 8]. Female sex, being Hispanic or Black, prior AIDS, CD4+ T-cell count <200 cells/mm^3^, and lower socioeconomic status increase the risk of weight gain [1, 9].

To date, bariatric surgery (BS), commonly sleeve gastrectomy (SG) and gastric bypass (GBP), is a durable intervention when diet and lifestyle modifications fail to achieve weight goals. BS improves obesity-related complications and overall health, with high rates of remission in obesity comorbidities seen in the general population [10]. Limited data are available on the benefits of BS for PWH.

BS significantly manipulates the anatomy and the environment within the gastrointestinal tract; this could profoundly impact the absorption and metabolism of orally administered medications including antiretrovirals [11, 12]. Current data on pharmacokinetics and the activity of antiretrovirals post-BS are limited by the heterogeneous nature of the published case reports or the small size of the studies, the short follow-up after surgery (6–12 months), and the investigation of older antiretrovirals that are no longer preferred treatment options [12]. The latter limitation is particularly important because people who initiate newer antiretrovirals, such as INSTIs and TAF, are experiencing significant weight gain, and there is no data on whether these regimens might interfere with weight reduction post-BS.

Therefore, we conducted a retrospective national cohort study in the Netherlands in PWH post-BS. The primary objectives were to evaluate the virologic response of different ART regimens up to 18 months post-BS and to assess weight reduction in PWH post-BS. Secondary objectives included the number of ART switches post-BS, the plasma concentrations of several antiretrovirals post-BS, and the impact of BS on the metabolic status in PWH. Medication usage was also compared before and 18 months after BS.

## METHODS

### Study Cohort

Eligible participants were selected from the Dutch AIDS Therapy evaluation in the Netherlands (ATHENA) cohort database for which data are routinely collected from all consenting PWH treated at 1 of the 24 Dutch HIV treating centers [13]. The ATHENA cohort is an ongoing open nationwide cohort, managed by Stichting HIV Monitoring, in which 98% of all PWH in care within the Netherlands are included. For the current analyses, we included PWH who were using any ART regimen and who underwent BS between January 2004 and January 2022. Patients for whom BS was performed prior to HIV diagnosis, who received only a gastric band, and whose BS and/or follow-up visits were performed outside the Netherlands were not included.

### Data Collection

Baseline was defined as the documented date of BS. For plasma HIV-1 RNA viral load (VL), the latest available measurement within ≤6 months prior to the BS date was considered baseline. HIV VL, CD4+ T-cell count, and treatment history were collected at baseline and at all available time points up to 18 months post-BS. Demographics including age, weight, BMI, blood pressure, low-density lipoprotein cholesterol (LDL-c), high-density lipoprotein cholesterol (HDL-c), triglycerides, and serum creatinine were collected at baseline and at 3 follow-up visits 6, 12, and 18 months (±2 months) post-BS, if available.

Medications were stratified as obesity-related medications (antihypertensive, antilipidemic, and antidiabetic) and total medications (eg, anticancer and antidepressants). Prescribed vitamin/mineral supplements were also retrieved from medication records.

### Plasma Concentrations of ART Post-BS

Results of therapeutic drug monitoring (TDM) were collected if monitoring was performed by individual physicians up to 18 months post-BS. TDM results were reported if they were collected at trough concentrations (≥16 hours post-intake for once-daily and ≥8 hours post-intake for twice-daily medications) to enable comparison with the minimum effective concentration of each drug at the end of the dosing interval. Values of minimum concentration targets of each drug were extracted from the related clinical trials.

### End Points

The primary end point was a confirmed virologic failure (VF) defined as 2 consecutive VL measurements ≥200 copies/mL up to 18 months post-BS. We also report the incidence of developing a single viral blip (detectable HIV-1 RNA of >40–200 copies/mL) after being virologically suppressed at baseline (VL <40).

BS efficacy was defined as a >20% loss of total body weight at 18 months post-BS. Currently, no definition exists for successful weight loss post-BS. However, the American Society for Metabolic and Bariatric Surgery has defined insufficient weight loss as <50% of excess weight loss or a corresponding <20% total body weight loss at 18 months post-BS [14]. The 20% weight loss was also used as a predictor for a complete remission of obesity-driven diabetes mellitus post-BS in the general population [15, 16].

The secondary end points for ART performance were the number of drug switches from the baseline ART regimen and the incidence of trough plasma concentrations below the minimum effective concentration for each antiretroviral with an available TDM. ART switches are defined as any addition to or deletion from the prescribed ART regimen at baseline.

The secondary outcome for BS efficacy was a significant improvement in the metabolic parameters including systolic and diastolic blood pressure, LDL-c and HDL-c, and creatinine clearance at 6, 12, and 18 months post-BS compared with baseline. Since there is no consensus on the improvement goals for metabolic parameters post-BS, we calculated the mean difference and statistical significance for each metabolic parameter before vs 6, 12, and 18 months after surgery. The number of obesity-related medications and the total number of medications were compared before and at 18 months after BS.

### Statistics

Virologic changes (VF or blips) and drug switches from the baseline ART regimen were described without statistical analysis due to the expected low number of events compared with the number of available ART regimens. Demographics with continuous variables were presented as medians and interquartile ranges (IQRs); n (%) was used for categorical data. A linear mixed-effects model for repeated measures was used to compare the mean differences and 90% confidence intervals (CIs) of the continuous metabolic parameters before vs 6, 12, and 18 months after BS (fixed effect). Patient number was set as a random effect. Due to the multiple testing performed on the continuous health parameters, we used a *P* value of .01 instead of .05 to correct for the increased rate of false-positives in multiple testing as previously recommended for disease-associated studies [17]. Data analysis and visualization were performed using SPSS (version 25, IBM Corp, Armonk, NY).

## RESULTS

Fifty-eight patients who underwent BS between January 2004 and January 2022 were identified; 7 were excluded (4 had a gastric band only, BS was performed prior to HIV diagnosis for 2, and 1 had BS and follow-up visits done outside the Netherlands (Supplementary Figure 1). Baseline data were available for the remaining 51 patients (Table 1).

**Table 1.** Baseline Data for the Selected Cohort Pre-Bariatric Surgery

Demographic	n (%) or Median (Interquartile Range)
Age, y	46 (37–52)
Gender	
Female	28 (55%)
Surgery type	
Gastric bypass	39 (76%)
Gastric sleeve	12 (24%)
Weight at BS, kg	121 (109–133)
Body mass index at BS, kg/m^2^	39.8 (37.7–43.8)
Time since HIV diagnosis, y	11 (7–16)
HIV-1 RNA <40 copies/mL^a^	43 (84%)
CD4+ T-cell count at BS, cells/mm^3^	820 (600–990)
Antiretroviral therapy at BS	
INSTI + 2 NRTIs	25 (49%)
NNRTI + 2 NRTIs	13 (25%)
PI + 2 NRTIs	5 (10%)
Others	5 (10%)
Unknown	3 (6%)

Abbreviations: BS; bariatric surgery; HIV, human immunodeficiency virus; INSTI, integrase strand transfer inhibitor; NNRTI, nonnucleotide reverse transcriptase inhibitor; NRTI, nucleotide reverse transfer inhibitor; PI, protease inhibitor.

^a^All patients with detectable viral load (VL) at surgery time had VL <200 copies/mL except 1 with 6000 copies/mL.

The median age at baseline was 46 years (IQR, 37–52), 55% were women, and 76% underwent GBP. Median weight (IQR) and BMI pre-BS were 121 kg (109–133) and 39.8 kg/m^2^ (37.7–43.8), respectively. Eighty-four percent (n = 43) of patients were virologically suppressed (plasma HIV-1 RNA <40 copies/mL) at the last measured VL prior to BS. The remaining patients (n = 8) had a detectable HIV-1 RNA level <200 copies/mL, except for 1 patient who had a VL of 6000 copies/mL before BS (ART regimen is unknown, CD4 = 810 T cells/mm^3^). At baseline, 49% of patients were on INSTI + 2 nucleo(s/t)ide reverse transcriptase inhibitors (NRTIs), and 29% used dolutegravir as an anchor drug. A full description of ART regimens used is provided in Supplementary Table 1.

### Virologic Outcomes Post-BS

Of 43 patients with full virologic suppression at baseline, 1 developed VF with 2 consecutive VL measurements of 487 and 420 copies/mL at month 8 post-BS (Table 2). This patient was a heavily pretreated woman with a history of VF on 4 previous regimens due to nonadherence. At the time of surgery (GBP), the patient was treated with an intensive regimen of twice-daily darunavir/ritonavir/raltegravir/maraviroc. Raltegravir was switched to dolutegravir. However, the VL fluctuated between undetectable and a repeat detectable HIV-1 RNA level that ranged from 75 to 1480 copies/mL in the first year post-BS. The patient had episodes of ART nonadherence before and after surgery. Resistance testing was not done at the time of VF. Resistance testing in the past showed extensive protease inhibitor (PI) resistance, but this was not relevant for darunavir/ritonavir. INSTI resistance was never tested.

**Table 2.** Virologic Events Up to 18 Months Post-Bariatric Surgery in Virally Suppressed Individuals at Baseline

Virologic event	Viral Load, copies/mL	ART Category	ART Regimen	BS Type	Time Post-BS, mo
Virologic failure^a^ >200 copies/mL (n = 1)	487–420	Others	DRV/r/RAL/MVC	Gastric bypass	8
Viral blip^a^ >40 copies/mL (n = 3)	80	PI + 2 NRTIs	TDF/FTC/DRV/r	Gastric bypass	6
	70	INSTI + 2 NRTIs	TDF/FTC/RAL	Gastric bypass	7
	49	NNRTI + 2 NRTIs	TDF/FTC/NVP	Gastric bypass	7

Abbreviations: ART, antiretroviral therapy; BS, bariatric surgery; DRV/r, ritonavir-boosted darunavir; FTC, emtricitabine; INSTI, integrase strand transfer inhibitor; MVC, maraviroc; NNRTI, nonnucleotide reverse transcriptase inhibitor; NRTIs, nucleotide reverse transfer inhibitor; NVP, nevirapine; PI, protease inhibitor; RAL, raltegravir; TDF, tenofovir disoproxil fumarate.

^a^Only reported if the patients were confirmed to have viral suppression prior to the event.

In 3 patients who had undetectable HIV-1 RNA at baseline, 3 viral blips occurred at 6, 7, and 7 months post-BS, respectively (all had undergone GBP). These patients were on different ART regimens that contained tenofovir disoproxil fumarate (TDF) and emtricitabine combined with nevirapine, raltegravir, or darunavir/ritonavir. Due to the low VL, no resistance testing was done on the blip samples. Previous resistance tests did not show PI resistance, and INSTI resistance was not tested. No VFs or viral blips were reported in the 12 patients who had SG in this cohort. None of the patients on dual therapy (n = 3) showed VF or blips, and their VL remained undetectable up to 18 months post-BS with good immune response (CD4 >500 T cells/mm^3^). There were no VF or blips detected in 3 patients who were on a dual ART regimen (dolutegravir plus lamivudine, n = 1, or darunavir/ritonavir, n = 2) and had SG. Four of 8 patients who had detectable VL pre-BS (VL >40 copies/mL) became fully suppressed by the end of the follow-up period; 4 patients had no VL by the end of the follow-up period including the patient with high VL at baseline (6000 copies/mL).

### Weight Reduction Post-BS

**Figure 1.**
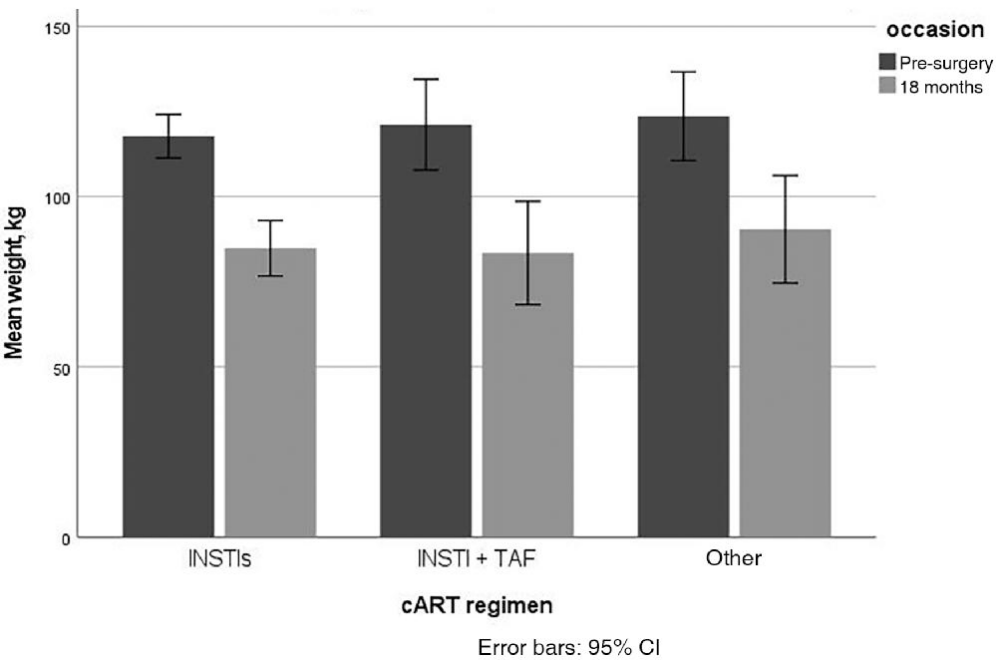
Mean weight per cART regimen at baseline and 18 months post-bariatric surgery. Abbreviations: cART, combination antiretroviral therapy; CI, confidence interval; INSTI, integrase strand transfer inhibitor; TAF, tenofovir alafenamide fumarate.

Eighty-nine percent of patients achieved >20% loss of baseline weight at 18 months post-BS, with a median percentage of weight loss (IQR) of 27% (21.4%–34.0%). Mean differences in weight were significantly lower at all post-BS time points compared with baseline (*P* < .001), with a mean difference of −33.5 (95% CI: −37.7 to −29.3) at 18 months post-BS. Interestingly, a group analysis showed no significant difference in weight reduction at 18 months among patients who continuously took INSTI without TAF, or INSTI plus TAF versus non-INSTI/non-TAF regimens in this cohort (*P* = .48 and .51, respectively; Figure 1).

**Figure 2.**
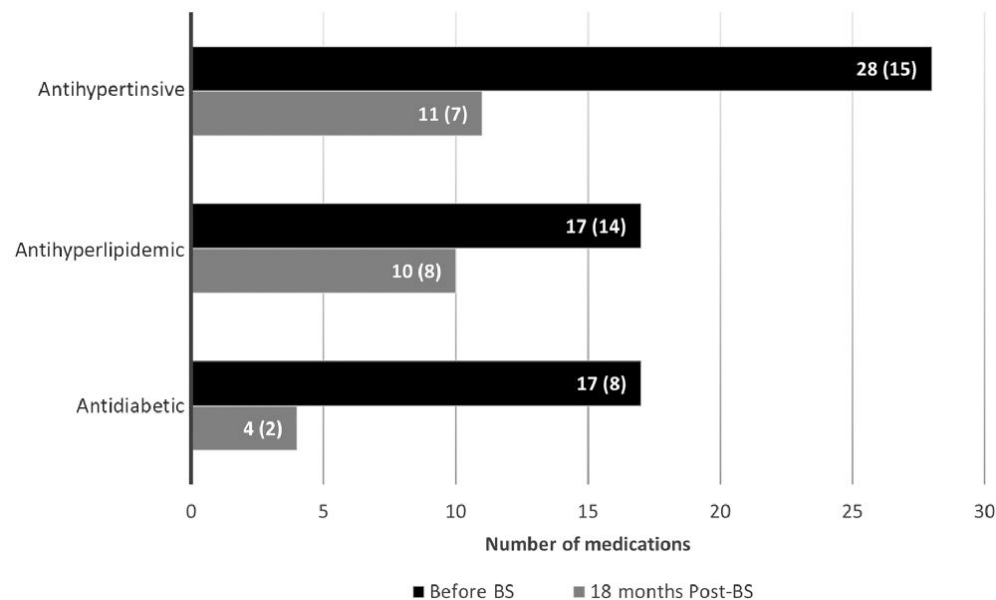
Number of obesity-related medications pre- and post-BS. Abbreviation: BS, bariatric surgery.

### ART Switches and Exposure Post-BS

There were 24 ART switches in 21 patients up to 18 months post-BS (Supplementary Table 2). The majority of switches were in the INSTI + 2 NRTIs group (n = 14, 58.3%), followed by NNRTI + 2 NRTIs (n = 4, 16.6%) and PI + 2 NRTIs (n = 2, 8.3%), which represents the same distribution of baseline ART class in the cohort. One switch was driven by VF (see Table 2). Another switch was driven by persistent low-level viremia post-GBP (40–119 copies/mL). Maraviroc was added to the regimen (TDF/emtricitabine/darunavir/ritonavir), and the VL was undetectable at the end of the follow-up period. An additional 2 switches were reported to be driven by low plasma levels of once-daily darunavir (+ cobicistat). Darunavir concentrations were 0.6 mg/L at 2 hours (post-SG) and 0.16 mg/L at 24 hours (post-GBP) with no reported VF. However, most ART switches were driven by routine non–BS-related reasons such as treatment simplification (n = 6, 25%), pregnancy (n = 3, 12.5%), or other (n = 5, 25%) including patient preference and availability of newer ART options. An overview of switching incidence and reasons is presented in Supplementary Table 2.

Thirteen plasma samples were collected at trough levels and taken within the first 4 months post-BS (Table 3). Levels of dolutegravir 50 mg, emtricitabine 200 mg, and tenofovir (formulated as TDF 245 mg or TAF 10 mg plus a booster) were similar or above the population levels at 24 hours with no predicted risk of subtherapeutic exposure below the targets of the minimum effective concentrations. However, there was controversy regarding plasma levels of once-daily darunavir/ritonavir 800/100 mg as 1 sample showed low exposure (0.13 mg/L) below the minimum effective concentration at 24 hours (0.20 mg/L), while the other 2 samples were within population levels. Plasma levels of elvitegravir were within the therapeutic concentrations. One elvitegravir concentration (0.17 mg/L) was at the margin of the minimum effective concentration (0.13 mg/L); nevertheless, it was still sufficient.

**Table 3.** Plasma Trough Concentrations for Antiretroviral Therapy Post-Bariatric Surgery

Antiretroviral	Dose^a^	Time Post- Bariatric Surgery, mo	Time Post-Intake, h	Plasma Level, mg/L	Viral Load at Therapeutic Drug Monitoring, copies/mL	Reference C_trough_ (Coefficient of Variation %), mg/L	Target C_trough_, mg/L	Interpretation
DRV^b^	800	1	16	3.30	116^c^	1.07 (33.6%) [18]	0.20 [19]	Increased exposure; no risk of subtherapeutic exposure
DRV^b^	800	4	18	3.51	<40			
DRV^b^	800	1	24	0.13	<40			Lower exposure; high risk of treatment failure
DTG	50	1	23	2.38	NA	1.20 (62%) [20]	0.32 [21]	Increased or adequate exposure; no risk of subtherapeutic exposure
DTG	50	4	23	1.05	<40			Adequate/therapeutic exposure
EVG^d^	150	1	16	0.48	<40	0.45 (41%) [22]	0.13 [22]	Adequate/therapeutic exposure
EVG^d^	150	1	22	0.17	<40			Adequate exposure with some risk in case of drug–drug interactions or other exposure-lowering factors
FTC	200	1	16	0.58	<40	0.09 (47%) [23]	NA	Increased or adequate exposure; no risk of subtherapeutic exposure
FTC	200	1	22	0.08	<40			
FTC	200	4	23	0.10	<40			
TNF (given as TDF)	245	4	23	0.08	<40	0.05 (25%) [24]	NA	Adequate/therapeutic exposure
TNF (given as TAF)	10	1	16	0.02	<40	0.01 [25]	NA	Adequate/therapeutic exposure
TNF (given as TAF)	10	1	22	0.01	<40			Adequate/therapeutic exposure

Abbreviations: DRV, darunavir; DTG, dolutegravir; EVG, elvitegravir; FTC, emtricitabine; NA, not available; TAF, tenofovir alafenamide fumarate; reference C_trough_, geometric mean of plasma concentration at the end of the dosing interval or 24 hours for once-daily dosing; target C_trough_, minimum effective concentrations at the end of the dosing interval or 24 hours for once-daily dosing; TDF, tenofovir disoproxil fumarate; TNF, tenofovir.

^a^All presented medications were given once daily.

^b^DRV boosted with ritonavir.

^c^This patient had detectable viral load pre-bariatric surgery at baseline.

^d^EVG was boosted with cobicistat.

### Metabolic Parameters and Medications Profiles

The lipid profiles (total cholesterol, LDL-c, and triglycerides) decreased significantly post-BS (*P* < .01; Table 4). Systolic blood pressure was lower post-BS compared with baseline; however, not to a statistically significant level. Changes from baseline were less apparent for HDL-c and serum creatinine.

**Table 4.** Metabolic Parameters Pre- and Post-Bariatric Surgery

Parameter	Baseline Mean (Standard Deviation)	Mean Difference From Baseline (95% Confidence Interval)					
		6 Months	*P* Value	12 Months	*P* Value	18 Months	*P* Value
Weight, kg	120.9 (19.9)	−26.7 (−29.4 to −24.1)	**<**.**001**	−32.0 (−35.1 to −28.9)	**<**.**001**	−33.5 (−37.7 to −29.3)	**<**.**001**
Body mass index, kg/m^2^	40.8 (5.5)	−8.8 (−9.7 to −8.0)	**<**.**001**	−10.9 (−11.9 to −9.9)	**<**.**001**	−11.1 (−12.7 to −9.7)	**<**.**001**
Systolic BP, mmHg	129.7 (13.1)	−3.7 (−9.4 to 1.8)	.2	−7.3 (−14.4 to −0.06)	.05 (0.048)	−5.7 (−15.2.8 to 3.7)	.2
Diastolic BP, mmHg	81.2 (8.8)	−2.9 (−6.7 to 0.9)	.1	−1.3 (−5.2 to 2.6)	.5	−3.9 (−8.1 to 0.3)	.06
High-density lipoprotein cholesterol, mmol/L	1.5 (2.1)	0.08 (−0.1 to 0.3)	.4	0.3 (−0.1 to 0.8)	.08	0.6 (−0.1 to 1.3)	.07
Low-density lipoprotein cholesterol, mmol/L	2.8 (0.84)	−0.5 (−0.8 to −0.2)	**<**.**01**	−0.5 (−0.9 to −0.06)	**<**.**01**	−0.7 (−0.9 to −0.4)	**<**.**01**
Total cholesterol, mmol/L	5.0 (0.2)	−0.8 (−1.1 to −0.5)	**<**.**001**	−0.8 (−1.3 to −0.2)	**<**.**01**	−0.9 (−1.3 to −0.4)	**<**.**001**
Triglycerides, mmol/L	2.5 (1.9)	−0.9 (−1.3 to −0.4)	**<**.**001**	−1.2 (−1.7 to −0.8)	**<**.**001**	−1.3 (−1.9 to −0.7)	**<**.**001**
Serum creatinine, µmol/L	73.9 (33.0)	2.0 (−7.3 to 11.3)	.7	2.4 (−7.4 to 12.3)	.6	2.0 (−9.3 to 13.4)	.7

*P* value <.01 are in bold.

Abbreviation: BP, blood pressure.

Post-BS, the total number of medications and obesity-related medications dropped from 203 to 103 and from 62 to 25, respectively (Supplementary Figure 1). Twelve patients stopped all of their non-HIV medications, approximately 50% stopped their antihypertensive or antihyperlipidemic medications, and the majority (75%) stopped their antidiabetic medications at 18 months post-BS (Figure 2). Thirty patients used vitamin/mineral supplements, and 17 used them at the end of the follow-up period. Separate intake is advised for patients, which is in line with the Dutch HIV treatment guidelines [26].

## DISCUSSION

This is the first cohort study on PWH who underwent BS for which virologic, pharmacologic, and metabolic parameters were analyzed. We did not detect insufficient virologic control in those with suppressed VL at the time of BS, and we did not detect multiple ART drugs with subtherapeutic plasma concentrations in this cohort post-BS. Additionally, the magnitude of weight loss and the decreased number of concomitant medications post-BS in PWH on ART was comparable to that observed in the general population [10].

The single VF seen in 1 individual was thought to be driven mainly by noncompliance with ART. Calcium carbonate was prescribed to this patient to prevent vitamin deficiency post-BS. Raltegravir is known to form a complex with divalent cations in mineral supplements, such as calcium, that prevents its absorption into the gastrointestinal tract. Nevertheless, this was not hypothesized to be the main driver of the VF in this patient due to their history of multiple VFs pre-BS. Moreover, the patient was on twice-daily raltegravir (400 mg), and this is known to compensate for the lower exposure to raltegravir when given with calcium carbonate compared with once-daily 1200 mg [27]. Generally, several therapeutic considerations, including dose adjustments and management of drug–drug interactions, should be maintained for patients on specific ART post-BS [11, 12].

Weight reduction was the major end point for BS efficacy in our study and was demonstrated to be high at 12 months with a slight increase at 18 months post-BS. These findings in PWH are consistent with data from the general population that demonstrate an accumulative weight loss up to 1 year after SG or GBP before weight loss plateaus or continues modestly [28–30]. Thus, a follow-up period of 18 months post-BS is considered suitable since the overall effect of BS on drug bioavailability and weight control is thought to be minimal after 1–1.5 years [28, 31]. Of note, BS efficacy is usually assessed clinically at 18 months post-BS to predict insufficient weight loss and/or the need for revision surgery [32]. In addition to BS, several pharmacologic interventions (ie, glucagon-like peptide 1 agonists) are emerging for long-term weight loss in the general population [33], with one ongoing study in PWH (Clinical Trials Registration, NCT04174755).

Our, albeit limited, results of plasma concentrations indicate that BS had no detrimental effect on elvitegravir, dolutegravir, emtricitabine, and tenofovir plasma concentrations. The latter 3 drugs are particularly important because their combination is listed as a preferred first-line treatment option in most HIV treatment guidelines. Two patients were switched post-BS due to expected low darunavir (+ cobicistat) plasma levels following SG or GBP (concentrations were not provided). Additionally, 2 of 3 trough plasma concentrations of darunavir/ritonavir in our analysis did not suggest a subtherapeutic exposure post-BS (Table 3). The available literature on darunavir concentrations post-BS is also contradictory, showing both similar (post-SG) [34] and decreased (post-SG and GBP) [35, 36] darunavir concentrations compared with population levels. It is noteworthy that darunavir should be taken with food for optimal absorption, which would not be achieved immediately post-BS due to restricted gastric capacity [12]. Although no literature suggests that darunavir results in a suboptimal viral control post-BS, our data support the need for additional research on darunavir exposure and the drivers of the pharmacokinetic variation post-BS. Furthermore, our findings suggest, for the first time, that once-daily TAF 10 mg (in combination with a booster) is probably sufficient for PWH who have undergone BS. Nevertheless, extensive plasma sampling is warranted for a comprehensive conclusion on TAF exposure post-BS.

Lipid measurements improved significantly post-BS in this cohort. Systolic blood pressure improved post-BS but not to a statistically significant level. Nevertheless, data on medication usage showed a 50% decrease in the use of antihypertensive medications in the same cohort during the same period. One explanation is the small sample size in our cohort, which might have hindered achieving the significance cutoff (*P* < .01) for blood pressure measurements. The overall decline in body weight and reduction in medication usage in our cohort is consistent with the outcomes of the GAstric Bypass to Treat obEse Patients With steAdy hYpertension (GATEWAY) randomized trial on obese patients with hypertension who underwent GBP. This trial reported significant reductions in LDL-c and triglycerides at 12 months post-GBP [10]. The study also reported a significant decrease in the number and dose of antihypertensive medications until the end of the 1-year follow-up period. We did not detect a trend toward improvement in creatinine clearance in PWH, which is similar to the GATEWAY data from the general population [10]. One possible explanation is the chronic use of several antiretrovirals associated with nephrotoxicity and decreased glomerular filtration, such as TDF and atazanavir, in our cohort [37]. Importantly, 40% of obesity-related medications (37 of 62) were stopped by the end of the follow-up period, which indicates an improved metabolic status despite the HIV- and ART-related pathophysiology. Simplifying medication usage in PWH is relevant as they present with higher polypharmacy and associated drug–drug interactions compared with populations not diagnosed with HIV [38–40].

The current analysis has some limitations. Although this is the largest analysis to date on PWH who underwent BS, the sample size was not sufficient for a comprehensive comparison of health improvement in PWH vs the general population. Furthermore, TDMs were not performed at VFs and viral blips, and available TDM samples did not involve all current antiretrovirals, including doravirine, atazanavir, and rilpivirine. The latter 2 have acid-dependent absorption and were considered not suitable post-BS on a theoretical basis with limited clinical data [12]. Medication intake was retrieved from patient files in which, for example, over-the-counter medications and vitamin supplements could be underreported. Additionally, drug adherence was not recorded in the current database, which complicates the interpretation of the virologic outcomes of ART in our study population. Nonetheless, the limited incidence of VFs and viral blips does not indicate major suboptimal adherence in our cohort. Finally, parameters such as C-reactive protein, plasma glucose, and glycated hemoglobin (HbA1c) might be obesity-relevant; however, they were not included in this analysis as they are not routinely collected for PWH during follow-up visits in the Netherlands.

Together, these initial data from a small cohort indicate that BS is a beneficial intervention in obese PWH using ART and that there is no clear worsening of virologic control. Weight and lipid profiles improved significantly post-BS in PWH. Caution should be used in people with a history of VF or ART nonadherence at the time of BS to avoid the risk of subtherapeutic exposure to ART and the subsequent poor virologic control.

## Supplementary Data

Supplementary materials are available at *Clinical Infectious Diseases* online. Consisting of data provided by the authors to benefit the reader, the posted materials are not copyedited and are the sole responsibility of the authors, so questions or comments should be addressed to the corresponding author.

## Supplementary Material

ciad404_Supplementary_Data

## Notes

***Financial support*.** There was no specific funding for this research. The ATHENA (AIDS Therapy evaluation in The Netherlands) cohort is managed by Stichting HIV Monitoring, which was granted funding from the Dutch Ministry of Health, Welfare and Sport through the Centre for Infectious Disease Control of the National Institute for Public Health and the Environment.

***Data availability***. Deidentified data used for the accepted manuscript will be made available on the Radboud data repository.
