# Supplementary material for: Outcomes of Bariatric Surgery in People With Human Immunodeficiency Virus: A Retrospective Analysis From the ATHENA Cohort
Source: Clin Infect Dis. 2023 Jul 1;77(11):1561–8. doi: 10.1093/cid/ciad404 (PMC10686945; doi:10.1093/cid/ciad404)
Supplement: ciad404_Supplementary_Data [file ciad404_supplementary_data.zip › Supplementary material_09062023_clean.docx]

**Supplementary material**

**Outcomes of bariatric surgery in people living with HIV: a retrospective analysis from ATHENA cohort**

Running title: **Virologic, metabolic and pharmacokinetic outcomes of antiretrovirals after bariatric surgery**

L Zino^1^, F Wit^2^, C Rokx^3^, JG den Hollander^4^, M van der Valk^2,5^, O Richel^6^, DM Burger^1^, A Colbers^1^

^1^Radboud University Medical Center, department of Pharmacy and Radboudumc Research Institute for Medical Innovation (RIMI), Nijmegen, The Netherlands

^2^Stichting HIV Monitoring, Amsterdam, the Netherlands

^3^Erasmus University Medical Center, Department of Medical Microbiology and Infectious Diseases and department of Internal Medicine, section of Infectious Diseases, Rotterdam, The Netherlands

^4^Maasstad ziekenhuis, Dept Internal Medicine & infectious Diseases Rotterdam, The Netherlands

^5^Amsterdam UMC, location University of Amsterdam, Infectious Diseases, Amsterdam Institute for Infectious diseases, Amsterdam, The Netherlands

^6^Radboud University Medical Center, Department of Infectious Disease and Radboudumc Research Institute for Medical Innovation (RIMI), Nijmegen, The Netherlands

Corresponding author:

Leena Zino, Radboud University Medical Center, department of Pharmacy and Radboudumc Research Institute for Medical Innovation (RIMI), Nijmegen, The Netherlands

E-mail address: Leena.zino@radboudumc.nl, telephone: +31243617744

Postal address: P.O. Box 9101, Geert Grooteplein-Zuid 10 (route 864), 6500 HB Nijmegen (864), The Netherlands

**Figure S1.**

Figure S1 Inclusion strategy of the METAL study

**Table S1.**

Table S1 Specifications of anchor drugs used at baseline

| **Anchor drug** | | **Frequency** |  |
| --- | --- | --- | --- |
| ATZ | 1 | | |
| BIC | 2 | | |
| DRV/r  DRV/c | 3  2 | | |
| DTG | 15 | | |
| DTG/DRV/r (dual therapy) | 1 | | |
| EFV | 3 | | |
| EVG/c | 6 | | |
| NVP | 10 | | |
| RAL | 3 | | |
| RAL/DRV | 3 | | |
| Unknown^ | 3 | | |
| **Total** | **51** | | |
|  |  | | |
| ATZ; atazanavir, BIC; bictegravir, DRV/r; ritonavir-boosted darunavir; DRV/c; cobicistat-boosted darunavir; EFV; efavirenz, EVG/c; cobicistat-boosted elvitegravir, FTC; emtricitabine; NVP; nevirapine, RAL; raltegravir. | | | |

**Table S2.**

Table S 2 Specifications of baseline cART that were switched post-BS

| **Reason** | **#** | **ART class** | **ART regimen** | **Time post-BS**  **(months)** |
| --- | --- | --- | --- | --- |
| Virologic failure | 1 | Others | DRV/r/RAL/MVC | 8 |
| Low level viremia | 1 | PI+ 2 NRTIs | TDF/FTC/DRV/r | 3 |
| Low plasma exposure | 2 | Others  PI+ 2 NRTIs | DRV/c/DTG  TAF/FTC/DRV/c | 2  2 |
| Simplification | 6 | INSTI+ 2 NRTIs  INSTI+ 2 NRTIs  PI+ 2 NRTIs  Others  PI+ 2 NRTIs  NNRTI+ 2 NRTIs | ZDV/3TC/RAL  TAF/FTC/EVG/c  TDF/FTC/ATV/r  RPV/DRV/r/RAL  TDF/FTC/DRV/r  TDF/FTC/NVP | 12  14  4  10  8  2 |
| Toxicity | 4 | PI+ 2 NRTIs (Nausea- dizziness)  PI+ 2 NRTIs (Increased alkaline phosphatase)  INSTI+ 2 NRTIs (Increased creatinine)  INSTI+ 2 NRTIs (Nausea-insomnia) | ABC/3TC/ATV/r  TDF/FTC/NVP  TDF/FTC/DTG  ABC/3TC/DTG | 16  7  10  4 |
| Pregnancy (wishes) | 3 | All INSTI+ 2 NRTIs | TAF/FTC/EVG/c  ABC/3TC/DTG  ABC/3TC/DTG | 11  14  15 |
| Other* | 6 |  |  |  |
| **Total** | **24** |  |  |  |
| *Other reasons were: availability of a newer ART candidate (n=3), patient preferences (n=2), or enrollment in a clinical trial with different regimen (n=1).  ABC, abacavir, ATZ/r; ritonavir-boosted atazanavir, BS; bariatric surgery, cART; combination antiretroviral therapy, DRV/r; ritonavir-boosted darunavir; DRV/c; cobicistat-boosted darunavir, DTG; dolutegravir, EVG/c; cobicistat-boosted elvitegravir, FTC; emtricitabine, INSTI; strand transfer integrase inhibitor, MVC; maraviroc, NRTIs; nucleotide reverse transfer inhibitor, NNRTI; non-nucleotide reverse transcriptase inhibitor, NVP; nevirapine, PI; protease inhibitor, RAL; raltegravir, RPV; rilpivirine, TAF; tenofovir alafenamide fumarate, TDF; tenofovir disoproxil fumarate, ZDV; zidovudine, 3TC; lamivudine. | | | | |

**Figure S2.**

Figure S2 Number of total medications pre- and post-BS
