# Supplementary figures and images for: Outcomes of Bariatric Surgery in People With Human Immunodeficiency Virus: A Retrospective Analysis From the ATHENA Cohort
Source: Clin Infect Dis. 2023 Jul 1;77(11):1561–8. doi: 10.1093/cid/ciad404 (PMC10686945; doi:10.1093/cid/ciad404)

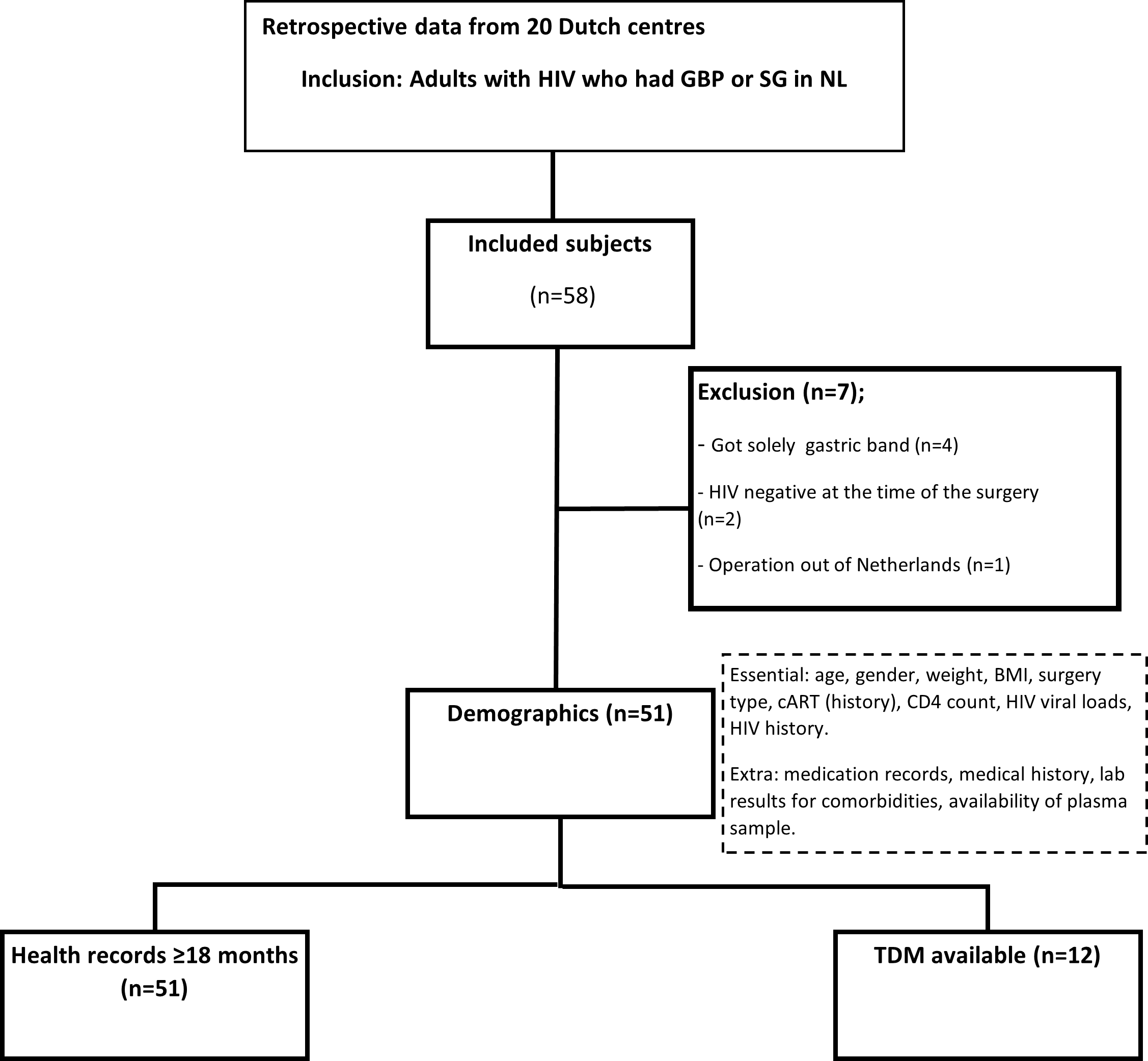

Supplement: ciad404_Supplementary_Data [file ciad404_supplementary_data.zip › Figure S1-kaye.tiff]

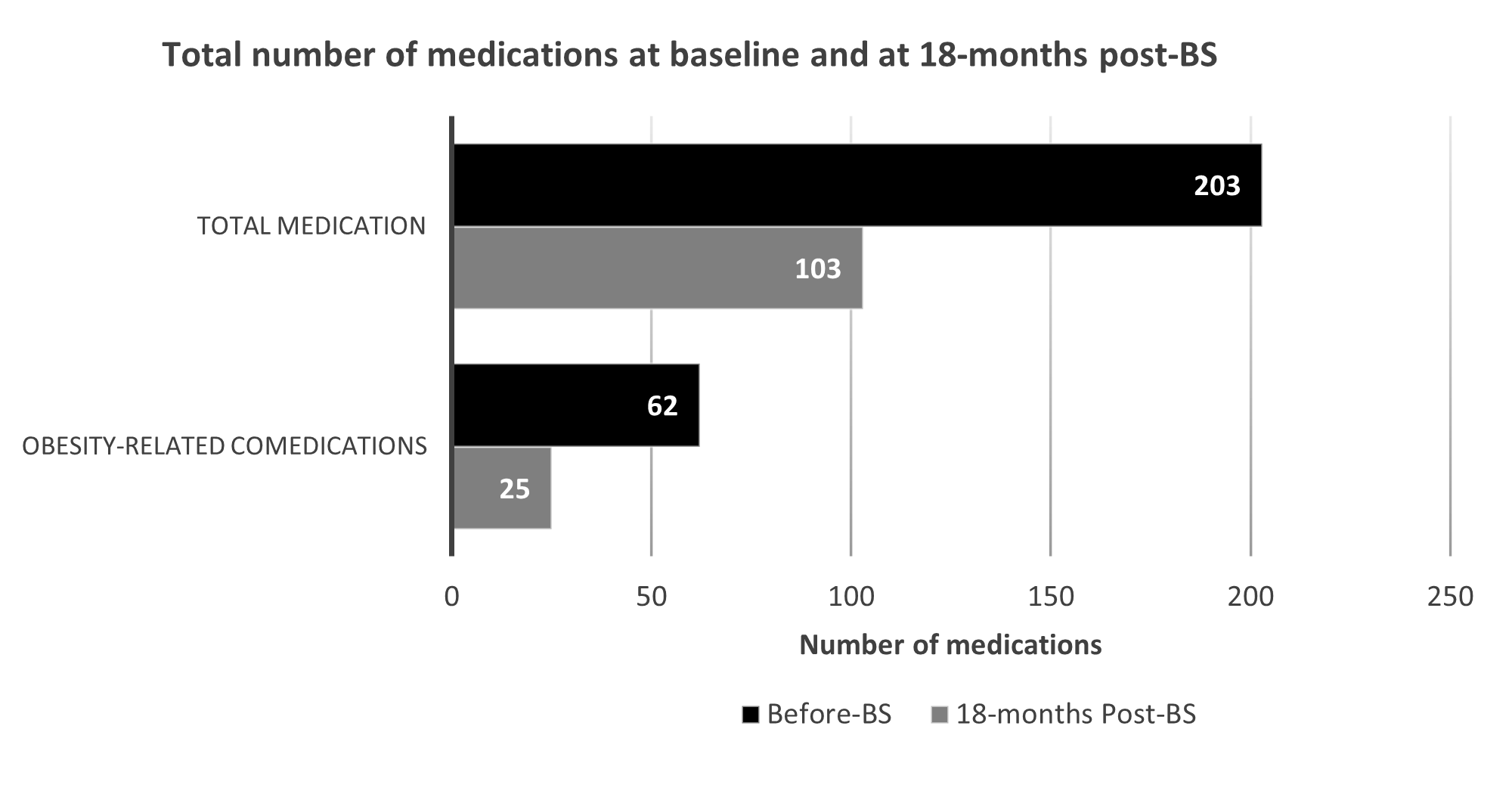

Supplement: ciad404_Supplementary_Data [file ciad404_supplementary_data.zip › figure S2.tif]
